# Self‐Perceived Need for Dental Treatment Among Brazilian Adolescent Students: Associations With Self‐Perceptions of Oral Health, Related Behaviours and Sociodemographic Factors

**DOI:** 10.1111/ipd.70018

**Published:** 2025-07-25

**Authors:** Leonardo Essado Rios, Maria do Carmo Matias Freire

**Affiliations:** ^1^ School Dental Health Service, Inhumas Campus Federal Institute of Goiás Brazil; ^2^ Postgraduate Program in Dentistry Federal University of Goiás Brazil

**Keywords:** adolescents, dental care, health risk behaviours, oral health, self‐perception, toothache

## Abstract

**Background:**

The need for dental treatment among adolescents can be assessed based on their self‐perception.

**Aim:**

To estimate the prevalence of self‐perceived dental treatment need (SPDTN) among adolescent students and associated factors.

**Methods:**

A cross‐sectional study was conducted in Midwest Brazil. The participants were adolescents (*N* = 3034) aged 13–19 from 14 public schools. Data were collected using self‐administered questionnaires. Adjusted odds ratios (OR) were estimated using binary logistic regression with a hierarchical approach.

**Results:**

SPDTN was reported by 41.4% of the sample. Older adolescents were more likely to have SPDTN than younger ones (OR = 1.14). Those who self‐rated their oral health negatively had 3.92 greater odds of having SPDTN than those who rated it positively. [Correction added on 27 September 2025, after first online publication: The sentence “Those who self‐rated their oral health negatively had 3.92 times greater odds of having SPDTN than those who rated it positively” was changed in this version.] SPDTN was directly associated with negative perceptions of dental appearance (OR = 2.97), chewing (OR = 1.80) and relationships with others affected by oral health (OR = 1.59). Moreover, SPDTN was associated with adolescents reporting toothache (OR = 1.78) and bleeding gums (OR = 1.41). High consumption of sweets and going to the dentist due to a toothache instead of periodic examinations increased the odds of SPDTN by 1.46 and 2.36, respectively.

**Conclusions:**

The prevalence of SPDTN among adolescents was high and associated with negative perceptions regarding their oral health, unhealthy behaviours and older age.

**Summary**

- Why this study is important to pediatric dentists
  ○ Adolescents' self‐perceived dental treatment need (SPDTN) is a crucial subjective indicator of unmet dental needs at this life course stage.
  ○ Little is known about the factors associated with adolescents' SPDTN since previous studies have focused primarily on comparing subjective and normative treatment needs.
  ○ Using a comprehensive hierarchical approach, this study highlights the main determinants of adolescents' SPDTN, which should be used to identify dental care needs among this age group.

## 1 Introduction

Self‐perception of oral health (SPOH) is how individuals evaluate their oral health subjectively [1, 2, 3]. SPOH's assessment can provide reliable information regarding oral health and has been associated with normative needs (i.e., those needs objectively diagnosed by dental professionals, using clinical criteria) [4, 5, 6, 7].

Adolescence is a critical period of the life course regarding several risk factors for oral health [8]. Studies on SPOH among adolescents are scarcer than those among adults and the elderly. Results indicate that negative perceptions of oral health during adolescence are associated with psychosocial, sociodemographic (SD) and contextual factors [4, 5, 6, 9, 10, 11, 12, 13, 14].

The three dimensions of SPOH usually investigated in previous studies among adolescents are self‐perception of oral health needs (SPOHN), self‐perception of the oral health itself expressed as self‐perceived oral symptoms (SPOS) and self‐perception of dental treatment need (SPDTN). SPOHN concerns the capability of individuals to report oral health needs (e.g., by self‐rating one or more of their oral‐related abilities, or their dental appearance, as well as by rating their overall oral health condition itself) [6, 7, 9, 13, 14]. SPOS concerns individuals' perception of symptoms related to their oral health (e.g., when they self‐report toothache or bleeding gums) [4, 6, 10, 14, 15, 16]. SPDTN indicates one's judgement about needing or not needing dental visits for professional treatment assistance [4, 5, 12, 15, 17, 18, 19, 20, 21].

As a subjective indicator of oral health, SPDTN assessments may be useful in identifying individuals or groups who need clinically defined dental treatment and oral health promotion interventions [4, 5, 15, 18]. However, the extant knowledge regarding the relationships between SPDTN and other dimensions of SPOH, as well as oral health‐related behaviours (OHRB) among adolescents, may be limited. To date, a single study has simultaneously evaluated the three dimensions of adolescents' SPOH, following a pre‐established model with SPDTN as the dependent variable and dental visits as the only OHRB among the independent variables [4]. Exploring the possible associations between the outcome SPDTN and other SPOH dimensions and OHRB, taking into consideration the adolescents' SD aspects, may provide a more comprehensive analysis. The results may be useful for planning oral healthcare strategies, integrating adolescents' perceptions of their needs as a proxy for clinical indicators. In this study, we aimed to estimate the prevalence of SPDTN among adolescent students in Midwest Brazil and its association with their SPOHN, SPOS, OHRB and SD factors.

## 2 Materials and Methods

A cross‐sectional study was conducted using data from a broader school‐based oral health survey. Self‐administered printed questionnaires were applied to adolescent students in all 14 campuses of a federal educational institution based in 13 municipalities in Goiás, Midwest Brazil. The World Bank classifies Brazil as an upper‐middle‐income country, and its Midwest region has one of the highest human development indexes in the country, according to official governmental agencies.

Sample size calculations were performed a priori for a set of variables of the research project using the OpenEpi website. As parameters for the present analysis, we used a hypothetical SPDTN frequency of 25.6% (the lowest percentage found in previous studies on this variable among adolescents) [15] and a sampling error of 2%. For a 95% confidence level, the estimated minimum sample size was 1826 individuals. To ensure the required sample size to cover all the intended analyses, plus a good safety margin, all the high school students aged 13 to 19 years were invited to participate.

The survey's questionnaire development was based on surveillance recommendations regarding self‐assessments of oral health and included questions from previous studies in Brazil and worldwide [22, 23, 24]. It was reviewed by a team of researchers with expertise in questionnaire surveys and pre‐tested on 14 adolescents. The questions included in the present analysis were about the adolescents' SD characteristics, SPOH and OHRB.

The dependent variable was SPDTN, assessed with a single question: Do you think you currently need dental treatment? Response categories: Yes/No/I do not know (adolescents who did not know and those who left the item blank were excluded, as in previous studies) [4, 19]. Independent variables were SPOHN, SPOS and SD factors, as well as the main OHRB described in scientific literature [25].

SPOHN was assessed with five questions on global and specific self‐assessment of oral health and its functions, as well as the impact perceived by the adolescents: (1) How would you rate your oral health at this point? (2) How would you rate the appearance of your teeth and gums? (3) How would you rate your chewing? (4) How would you rate your speech due to your teeth and gums? (5) How does your oral health affect your relationship with others? Response categories: Negative/Positive perception.

SPOS was assessed with two questions: (1) During the past 6 months, did you have a toothache? (Not counting the pain caused by braces, if you use them). (2) Do your gums bleed when you brush? Response categories: yes/no.

OHRB variables were as follows: Frequency of daily toothbrushing (less than twice/twice or more); sweets consumption during the last 7 days (high consumption: 5 days or more/low consumption: 4 days or less) [26]; soft drinks consumption during the last 7 days (high consumption: 5 days or more/low consumption: 4 days or less) [26]; dental attendance—past 12 months (no visit/once or more); dental attendance—usual motivation (only in case of toothache/periodically for check‐ups); and smoking—past 30 days (yes/no); second‐hand smoking—past 7 days (yes/no); and alcohol use—past 30 days (yes/no).

SD factors were as follows: sex (male/female); self‐reported colour or race based on the frequencies of SPDTN (black and Indigenous/yellow, brown and white); age, in years; and maternal education level used as a proxy for socioeconomic position (low: 8 years of study or less/high: 9 years of study or more).

The data were analysed using the IBM SPSS software (Version 21). For descriptive statistics, the absolute number (*N*) and relative frequencies (%) were calculated, or the mean value (M) and standard deviation (SD) (continuous variable age). Bivariate associations between the study variables were assessed using the Chi‐square test (categorical variables) or the independent‐samples *t*‐test (continuous variable age). The statistical significance level was *α* = 0.05.

Logistic regression was performed to identify the independent variables associated with the dependent variable (SPDTN, category yes). Odds ratios (OR) and 95% Confidence intervals (CI) were estimated, using the block entry method in the multivariate model. The cutoff point to include variables in the unadjusted regression model was a *p* < 0.20 obtained in the previous bivariate analyses. In the final regression models, only variables statistically associated (*p* < 0.05) with SPDTN were maintained in unadjusted and adjusted tests.

**FIGURE 1:**
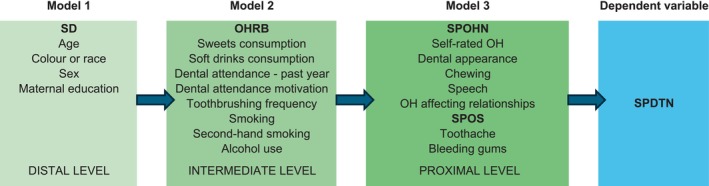
A hierarchical approach to Brazilian adolescent students' self‐perceived need for dental treatment and associated factors. OHRB, oral health‐related behaviours; OH, oral health; SD, sociodemographic factors; SPDTN, self‐perceived dental treatment need; SPOHN, self‐perceived oral health needs; SPOS, self‐perceived oral symptoms.

A hierarchical approach was used for variable selection and modelling (Figure 1). Three levels were incorporated into the model: a distal level that included SD variables (Level 1), an intermediate level containing OHRB variables (Level 2) and a proximal level that included the SPOHN and SPOS variables (Level 3).

The study was approved by the Research Ethics Committee of the Federal University of Goiás (REC/UFG) (Approval N. 2142027) and the Federal Institute of Goiás (Approval N. 2556510). We ensured the participants' rights to anonymity, confidentiality and to stop participating at any time with no adverse effects whatsoever. Adolescents who freely agreed to take part signed a free and informed consent. The study was exempt from requiring permission from parents/guardians of those aged under 18.

## 3 Results

Among the 3043 invited students, 3034 agreed to participate in the study (response rate = 99.7%). More than half of the participants were female (53.6%), aged between 16 and 19 (63.2%). Their self‐reported colour or race was mainly brown (50.9%), followed by white (31.4%), black (13.1%), yellow (3.8%) and Indigenous (0.5%). Most of their mothers had a high level of education (69.2%).

Current dental treatment need (SPDTN) was reported by 41.4% of the students. Regarding SPOHN, nearly one‐third had negative perceptions of their oral health (28.2%), appearance of teeth and gums (30.3%) and chewing (30.1%). About 13.0% presented a negative assessment of their speech due to teeth and gums, and 25.3% considered that their oral health negatively affected their relationships with others. As to SPOS variables, toothache in the past 6 months and gum bleeding were reported by one‐third and almost 40.0% of the participants, respectively (Table 1).

**TABLE 1:** Self‐perceptions of dental treatment need, oral health needs and oral health symptoms among adolescent students in Midwest Brazil (*N* = 3034).

Self‐perception of dental treatment need	*N*	%
Do you think you currently need dental treatment?		
Yes	1255	41.4
No	1048	34.5
Didn't know	707	23,3
No response	24	0.8
**Self‐perception of oral health needs**		
How would you classify your oral health at this point?		
Negative (regular, bad, very bad)	856	28.2
Positive (good, very good)	2108	69.5
Didn't know	52	1.7
No response	18	0.6
How would you classify the appearance of your teeth and gums?		
Negative (regular, bad, very bad)	920	30.3
Positive (good, very good)	2061	67.9
Didn't know	37	1.2
No response	16	0.5
How would you classify your chewing?		
Negative (Regular, bad, very bad)	913	30.1
Positive (Good, very good)	2034	67.0
Didn't know	71	2.3
No response	16	0.5
How would you classify your speech due to your teeth and gums?		
Negative (Regular, bad, very bad)	392	12.9
Positive (Good, very good)	2541	83.8
Didn't know	85	2.8
No response	16	0.5
How does your oral health affect your relationship with others?		
Negative (Affects, a little to a lot)	767	25.3
Positive (Does not affect)	1861	61.3
Didn't know	389	12.8
No response	17	0.6
**Self‐perception of oral health symptoms**		
In the past 6 months, have you had a toothache? (not counting the pain caused by braces, if you use them)		
Yes (a little to a lot of pain)	920	30.3
No	1644	54.2
Didn't know/remember	455	15.0
No response	15	0.5
Do your gums bleed when you brush?		
Yes (sometimes to always)	1171	38.6
No	1848	60.9
No response	15	0.5

Most adolescents used to brush their teeth twice or more a day (91.7%) and consumed sweets (51.7%) and soft drinks (79.3%) for 4 days or less during the past 7 days. More than half had visited the dentist in the past year (56%), and their usual motivation was mainly for periodical check‐ups (38.3%). Almost 8.0% were smokers, and more than half (55.5%) had been exposed to second‐hand smoke during the past 7 days. Around four out of every 10 adolescents had consumed alcoholic beverages in the past 30 days (Table 2).

**TABLE 2:** Oral health‐related behaviours among adolescent students in Midwest Brazil (*N* = 3034).

Oral health‐related behaviours	*N*	%
In the past 30 days, how many times a day have you usually brushed your teeth?		
Less than two	235	7.7
Two or more	2781	91.7
No response	18	0.6
In the past 7 days, how many days did you eat sweets? (candies, chewing gum, chocolates, lollipops, etc.)		
5 days or more	1456	48.0
4 days or less	1569	51.7
No response	9	0.3
In the past 7 days, how many days did you drink soft drinks?		
5 days or more	621	20.5
4 days or less	2407	79.3
No response	6	0.2
In the past 12 months, how many times have you been to the dentist? (not counting visits for the maintenance of braces, if you use them)		
None	1314	43.3
Once or more	1700	56.0
No response	20	0.7
What usually makes you go to the dentist? (not counting visits for the maintenance of braces, if you use them)		
I only go when I have a toothache	1060	34.9
I go periodically for check‐ups	1162	38.3
Never been to the dentist	205	6.8
Didn't know/remember	586	19.3
No response	21	0.7
Currently, do you smoke cigarettes? (select yes if you have smoked at least one cigarette in the past 30 days)		
Yes	241	7.9
No	2787	91.9
No response	6	0.2
In the past 7 days, how many days has anyone smoked in your presence?		
Once or more	1685	55.5
None	1335	44.0
No response	14	0.5
In the past 30 days, how many days have you had at least a glass or a dose of alcohol? (one dose is equivalent to a can of beer, cachaça, whiskey, etc.)		
Once or more	1151	37.9
None	1874	61.8
No response	9	0.3

The results of the bivariate associations between SPDTN and each independent variable are in Table 3. All the SPOHN and SPOS variables were associated with SPDTN (*p* < 0.001). Among the OHRB, the following were associated with SPDTN: low toothbrushing daily frequency (*p* = 0.030), high consumption of sweets (*p* = 0.005), dental visit in the last year (*p* < 0.001) motivated by periodical check‐ups (*p* < 0.001) and second‐hand smoking (*p* = 0.005). Among the SD variables, age (*p* < 0.001) and colour/race (*p* = 0.044) were associated with SPDTN.

**TABLE 3:** Characteristics of adolescent students in Midwest Brazil according to their self‐perceived dental treatment need (*N* = 2303).

Independent variables*	Self‐perceived dental treatment need				*p* **
	Do you think you currently need dental treatment?				
	Yes		No		
Self‐perception of oral health needs	*N*	%	*N*	%	
How would you classify your oral health at this point? (*N* = 2267)					
Negative (regular, bad, very bad)	610	50.0	78	7.5	< 0.001
Positive (good, very good)	611	50.0	968	92.5	
How would you classify the appearance of your teeth and gums? (*N* = 2277)					
Negative (regular, bad, very bad)	603	48.7	116	11.2	< 0.001
Positive (good, very good)	634	51.3	924	88.8	
How would you classify your chewing? (*N* = 2256)					
Negative (regular, bad, very bad)	525	42.8	188	18.3	< 0.001
Positive (good, very good)	702	57.2	841	81.7	
How would you classify your speech due to your teeth and gums? (*N* = 2239)					
Negative (regular, bad, very bad)	243	20.1	76	7.4	< 0.001
Positive (good, very good)	968	79.9	952	92.6	
How does your oral health affect your relationship with others? (*N* = 2035)					
Negative (it affects, a little to a lot)	429	39.0	189	20.0	< 0.001
Positive (it does not affect)	664	61.0	758	80.0	
**Self‐perception of oral health symptoms**					
In the past 6 months, have you had a toothache? (not counting the pain caused by braces, if you use them) (*N* = 1996)					
Yes (a little to a lot of pain)	508	47.8	213	22.8	< 0.001
No	555	52.2	720	77.2	
Do your gums bleed when you brush? (*N* = 2300)					
Yes (sometimes to always)	575	45.9	325	31.1	< 0.001
No	679	54.1	721	68.9	
**Oral health‐related behaviours**					
In the past 30 days, how many times a day have you usually brushed your teeth? (*N* = 2298)					
Less than two	108	8.6	65	6.2	0.030
Two or more	1145	91.4	980	93.8	
In the past 7 days, how many days did you eat sweets? (candies, chewing gum, chocolates, lollipops, etc.) (*N* = 2297)					
5 days or more (high consumption)	638	51.0	471	45.1	0.005
4 days or less (low consumption)	614	49.0	574	54.9	
In the past 7 days, how many days did you drink soft drinks? (*N* = 2300)					
5 days or more (high consumption)	274	21.9	213	20.4	0.385
4 days or less (low consumption)	980	78.1	833	79.6	
In the past 12 months, how many times have you been to the dentist? (Not counting visits for the maintenance of braces if you use them.) (*N* = 2298)					
None	598	47.7	365	34.9	< 0.001
Once or more	655	52.3	680	65.1	
What usually makes you go to the dentist? (Not counting visits for the maintenance of braces if you use them.) (*N* = 1736)					
I only go when I have a toothache	502	56.8	303	35.6	< 0.001
I go periodically for check‐ups	382	43.2	549	64.4	
Currently, do you smoke cigarettes? (Select yes if you have smoked at least one cigarette in the past 30 days.) (*N* = 2298)					
Yes	97	7.7	95	9.1	0.250
No	1155	92.3	951	90.9	
In the past 7 days, how many days has anyone smoked in your presence? (*N* = 2292)					
Once or more	737	59.0	553	53.1	0.005
None	513	41.0	489	46.9	
In the past 30 days, how many days have you had at least a glass or a dose of alcohol? (One dose is equivalent to a can of beer, cachaça, whiskey, etc.) (*N* = 2298)					
Once or more	483	38.5	395	37.8	0.713
None	770	61.5	650	62.2	
**Sociodemographic factors**					
Mean age (SD) (*N* = 2293)	16.1	1.11	15.9	1.08	< 0.001
Sex (*N* = 2300)					
Male	569	45.4	505	48.2	0.190
Female	683	54.6	543	51.8	
Colour or race (*N* = 2296)					
Black/Indigenous	193	15.5	131	12.5	0.044
Yellow/Brown/White	1056	84.5	916	87.5	
Maternal education (*N* = 2221)					
Low (≤ 8 years of study)	348	28.7	257	25.5	0.092
High (≥ 9 years of study)	865	71.3	751	74.5	

Abbreviation: SD, standard deviation.

* A smaller number of cases in the covariates indicates missing data and/or responses ‘I don't know/remember’.

** Categorical independent variables: Pearson's chi square test; Numerical independent variable (age): independent samples *t*‐test.

The following independent variables were associated with SPDTN only in the unadjusted regression analysis, losing statistical significance in the adjusted model: (1) In the distal level, colour/race (OR = 1.28; CI = 1.07–1.62). (2) In the intermediary level, frequency of toothbrushing (OR = 1.42; CI = 1.03–1.96), and dental attendance in the last year (OR = 1.70; CI = 1.44–2.01); (3) In the proximal level, speech due to teeth and gums (OR = 3.15; CI = 2.39–4.13) (Table 4).

**TABLE 4:** Logistic regression of factors associated with self‐perceived dental treatment need among adolescent students in Midwest Brazil (*N* = 2303).

Independent variables	Self‐perceived dental treatment need (yes)			
	Unadjusted		Adjusted	
Level 1—sociodemographic characteristics	OR	95% CI	OR	95% CI
Age (continuous)	1.14	1.06–1.23*	1.14	1.06–1.23***
Sex				
Male	0.90	0.76–1.06		
Female	1			
Colour or race				
Black/Indigenous	1.28	1.07–1.62**		
Yellow/Brown/White	1			
Maternal education				
Low (≤ 8 years of study)	1.18	0.97–1.42		
High (≥ 9 years of study)	1			
Level 2—oral health‐related behaviours^a^				
Daily toothbrushing frequency				
Less than two times	1.42	1.03–1.96**		
Two times or more	1			
Sweets consumption (past week)				
High (≥ 5 days)	1.27	1.07–1.49***	1.46	1.20–1.77*
Low (≤ 4 days)	1		1	
Dental attendance (past year)				
No	1.70	1.44–2.01*		
Yes	1			
Dental attendance motivation				
Toothache	2.38	1.96–2.89*	2.36	1.94–2.87*
Periodical check‐ups	1		1	
Second‐hand smoking				
Yes	1.27	1.08–1.50***		
No	1			
Level 3—self‐perception of oral health needs and symptoms^b^				
Self‐rated oral health				
Negative	12.39	9.59–16.01*	3.92	2.63–5.82*
Positive	1		1	
Appearance of teeth and gums				
Negative	7.58	6.06–9.47*	2.97	2.03–4.34*
Positive	1		1	
Chewing				
Negative	3.35	2.75–4.06*	1.80	1.32–2.46*
Positive	1		1	
Speech due to teeth and gums				
Negative	3.15	2.39–4.13*		
Positive	1			
Relationships affected by oral health				
Negative	2.56	2.10–3.13*	1.59	1.18–2.13***
Positive	1		1	
Toothache (past 6 months)				
Yes	3.09	2.55–3.76*	1.78	1.34–2.35*
No	1		1	
Gum bleeding during toothbrushing				
Yes	1.88	1.58–2.23*	1.41	1.08–1.84**
No	1		1	

Abbreviations: CI, confidence intervals; OR, odds ratio.

^a^ Adjusted for age.

^b^ Adjusted for sweets consumption, dental attendance motivation and age. Statistical significance (Wald chi‐square).

* *p* < 0.001.

** *p* < 0.05.

*** *p* < 0.01.

The results of the adjusted models are in Table 4. In the distal level (model 1), a direct association between SPDTN and age was observed (OR = 1.14; CI = 1.06–1.23). Among OHRB variables (intermediate level/model 2), adolescents who reported high consumption of sweets in the past week were more likely to have SPDTN, compared to those who reported low consumption (OR = 1.46; CI = 1.20–1.77). SPDTN was also associated with dental attendance motivated by toothache, compared to dental attendance motivated by periodical check‐ups (OR = 2.36; CI = 1.94–2.87). In the proximal level (model 3), adolescents who self‐rated their oral health negatively were more likely to perceive that they needed dental treatment (OR = 3.92; CI = 2.63–5.82), compared with those who self‐rated it positively. Also, SPDTN was directly associated with negative perceptions of the appearance of teeth and gums (OR = 2.97; CI = 2.03–4.34), chewing (OR = 1.80; CI = 1.32–2.46) and relationships affected by oral health (OR = 1.59; CI = 1.18–2.13). Regarding SPOS variables, those who reported toothache in the past 6 months and gum bleeding during brushing had 1.78 (CI = 1.34–2.35) and 1.41 (CI = 1.08–1.84) greater odds to perceive dental treatment need, respectively, compared with ones who did not report such symptoms.

## 4 Discussion

Estimating the extent to which the adolescents' SPDTN is associated with other self‐perception measures, behaviours and individual characteristics might contribute to drawing a profile of those with unmet dental needs. Results of this study showed a high prevalence of SPDTN among Brazilian adolescent students. Moreover, using a hierarchical approach, associations were found between SPDTN and SPOHN, SPOS, as well as OHRB and SD characteristics. Existing research on the relationships between the three dimensions of SPOH among adolescents and their OHRB is scarce [4]. Therefore, our findings expand the knowledge about the aspects related to SPDTN, indicating other variables that may influence how adolescents perceive their oral health.

Previous studies reported prevalence rates of adolescents' SPDTN ranging from 25.6% to 88.6%. In our sample, the prevalence (41.4%) was lower compared to other studies in Brazil [4, 5, 11, 12, 17, 21]. Moreover, it was comparable to the proportion found in Tanzanian [20] and higher than among American adolescents [15]. These discrepancies may be attributed to the subjective nature of the variable and cultural differences, but may also indicate oral health‐related regional inequalities in Brazil and globally.

Adolescents' SPDTN was directly associated with their SPOHN, mainly by negative ratings of the overall oral health status and dental appearance. To a lesser extent, it was associated with their negative perceptions of chewing ability and relationships with others. In the same direction, the two variables analysed in the study regarding the SPOS dimension, toothache and gum bleeding, were moderately associated with SPDTN. Our findings corroborate previous studies that observed direct associations between SPDTN among adolescents and the occurrence of oral symptoms [4, 11, 12, 15, 17]. However, the global oral status and aesthetic conditions related to teeth and gums had a greater influence on adolescents' SPDTN, unlike the study among American adolescents, who based their negative assessments mainly on oral symptoms [15].

Another variable associated with SPDTN was the adolescent's self‐perceived difficulty in chewing, corroborating a study on Tanzanian adolescents [20]. The associations of tooth decay and loss with SPDTN have been documented [5, 11], which may contribute to elucidating this finding. Eating‐related abilities represent an important indicator when assessing subjective treatment needs in this age group. Furthermore, it is noteworthy that difficulty in chewing is also associated with adults' SPDTN [27].

This was the first study reporting an association between adolescents' SPDTN and high consumption of sweets. The association between sweet consumption and dental caries is well‐established [28]. Thus, sweets consumption appears to be an important complementary behavioural indicator to the subjective assessment of the need for dental treatment among this age group. Moreover, the adolescents' SPDTN was directly associated with their usual motivation to seek restorative rather than preventive dental care, as reported in previous studies among Brazilian adolescents [4, 29]. This symptomatic pattern for seeking dental care among adolescents could be changed through oral health education to stimulate preventive dental appointments [11, 12]. Besides, expanding access to oral healthcare services may be considered of utmost importance [20].

Regarding the SD factors analysed in this study, SPDTN was only associated with an increased age. It has been suggested that older adolescents are in a more advanced process of developing critical awareness regarding their oral health, compared to the younger ones [30]. Therefore, they may be more concerned about aesthetics and the impact of their oral health on their self‐esteem and socialisation, which may lead to a greater perception of the need for treatment [17, 30].

We recommend that dental services for adolescent students in public schools seek to encompass their self‐perception of oral health. Based on the present findings, priority should be given to those who report SPDTN and have a negative self‐perception of oral health, chewing and appearance that is affecting their relationships, those who have had a recent toothache and bleeding gums when brushing their teeth and those with a high frequency of sweets consumption who usually visit the dentist only in case of toothache.

As this is a cross‐sectional analytical study, the findings do not allow for causal inferences or statements about temporal relationships between variables. The present data are representative of adolescents in Midwest Brazil, and external validity may not apply to adolescents studying in private schools. Another limitation of this study is the lack of reliability tests of the questionnaire. Also, the substantial exclusion of a large proportion of data in bivariate and multivariate analyses, mainly due to responses I do not know/remember, should be pointed out, as well as the potential social desirability bias of adolescent lifestyle‐related self‐reported data. Regarding strengths, we highlight the study rationale, methodological aspects regarding large sample size, high response rate and comprehensive analysis including the three main dimensions of SPOH and the main OHRB among adolescents.

To conclude, the prevalence of SPDTN among the adolescent students investigated was high and associated with their negative perceptions regarding SPOHN and SPOS, unhealthy OHRB and older age. We suggest these findings should be considered for planning oral health services aimed at this age group and decision‐making regarding which criteria to adopt to prioritise care for adolescents based on their self‐perception. This may help ensure priority to those most in need, with the potential to promote equity in oral health strategies targeting adolescent students.

## Author Contributions

L.E.R. developed the research question and objective of the study, collected and prepared the data, performed the statistical analysis and wrote the manuscript. M.C.M.F. guided the project development, supervised the study, and was involved in writing and reviewing the manuscript.

## Conflicts of Interest

The authors declare no conflicts of interest.

## Data Availability

The data that support the findings of this study are available from the corresponding author upon reasonable request.

## References

[1] K. A. Atchison and H. C. Gift , “Perceived Oral Health in a Diverse Sample,” Advances in Dental Research 11, no. 2 (1997): 272–280, 10.1177/08959374970110021001. 9549993

[2] H. C. Gift , K. A. Atchison , and T. F. Drury , “Perceptions of the Natural Dentition in the Context of Multiple Variables,” Journal of Dental Research 77, no. 7 (1998): 1529–1538, 10.1177/00220345980770070801. 9663438

[3] D. Locker , M. Clarke , and B. Payne , “Self‐Perceived Oral Health Status, Psychological Well‐Being, and Life Satisfaction in an Older Adult Population,” Journal of Dental Research 79, no. 4 (2000): 970–975, 10.1177/00220345000790041301. 10831100

[4] I. P. Cunha , F. L. Mialhe , A. C. Pereira , et al., “Self‐Perceived Dental Treatment Need Among Adolescents: A Hierarchical Analysis,” Community Dentistry and Oral Epidemiology 48, no. 2 (2020): 130–136, 10.1111/cdoe.12510. 31828838

[5] F. C. L. Delela , A. B. Martins , H. C. Ely , and C. Abegg , “Association Between Normative and Self‐Perceived Dental Treatment Need in Brazilian Students,” Journal of Oral Hygiene and Health 9, no. S4 (2021): 001, 10.4172/2332-0702.1000001.

[6] M. Nascimento , F. Cunha Soares , G. Dahllöf , G. Burgos Souto Maior , T. Kvist , and V. Colares , “Determinants of Self‐Perceived Oral Health in Adolescents: A Cross‐Sectional Study,” International Journal of Paediatric Dentistry 31, no. 2 (2021): 254–261, 10.1111/ipd.12664. 32419168

[7] S. A. Santos , F. R. Ortiz , B. A. Agostini , and T. M. Ardenghi , “Self‐Reported Oral Health and Normative Indices of Dental Caries Among Adolescents: A Cohort Study,” Brazilian Oral Research 36 (2022): e021, 10.1590/1807-3107bor-2022.vol36.0021. 35170689

[8] A. A. Lorente , V. S. López , A. P. Pardo , S. G. Pina , and O. C. Lillo , “Salud Oral: Influencia de los Estilos de Vida en Adolescentes,” Revista Pediatría de Atención Primaria 22 (2020): 251–261.

[9] M. L. B. Fagundes , O. L. D. Amaral Júnior , G. R. Menegazzo , et al., “Pathways of Socioeconomic Inequalities in Self‐Perceived Oral Health,” Brazilian Oral Research 36 (2022): e088, 10.1590/1807-3107bor-2022.vol36.0088. 35703713

[10] F. B. Lawal and E. B. Dosumu , “Self‐Reported and Clinically Evident Gingival Bleeding and Impact on Oral Health‐Related Quality of Life in Young Adolescents: A Comparative Study,” Malawi Medical Journal 33, no. 2 (2021): 121–126, 10.4314/mmj.v33i2.7. 34777707 PMC8560349

[11] R. T. Lopes , É. T. B. Neves , L. da Costa Dutra , et al., “Individual and Contextual Factors Associated With Adolescents' Self‐Perceived Need for Treatment,” International Journal of Environmental Research and Public Health 21, no. 4 (2024): 395, 10.3390/ijerph21040395. 38673308 PMC11049991

[12] I. M. L. F. Prata , A. F. Granville‐Garcia , É. T. B. Neves , et al., “Family Cohesion Is Associated With the Self‐Perceived Need for Dental Treatment Among Adolescents,” BioMed Research International 2021 (2021): 4504030, 10.1155/2021/4504030. 34631881 PMC8494560

[13] M. P. Silva , M. V. Vettore , M. A. B. Rebelo , et al., “Clinical Consequences of Untreated Dental Caries, Individual Characteristics, and Environmental Factors on Self‐Reported Oral Health Measures in Adolescents: A Follow‐Up Prevalence Study,” Caries Research 54, no. 2 (2020): 176–184, 10.1159/000506438. 32294648

[14] M. V. Vettore , S. F. H. Ahmad , C. Machuca , and H. Fontanini , “Socio‐Economic Status, Social Support, Social Network, Dental Status, and Oral Health Reported Outcomes in Adolescents,” European Journal of Oral Sciences 127, no. 2 (2019): 139–146, 10.1111/eos.12605. 30648760

[15] R. J. Weyant , M. Manz , P. Corby , L. Rustveld , and J. Close , “Factors Associated With Parents' and Adolescents' Perceptions of Oral Health and Need for Dental Treatment,” Community Dentistry and Oral Epidemiology 35, no. 5 (2007): 321–330, 10.1111/j.1600-0528.2006.00336.x. 17822480

[16] P. Nadanovsky , A. P. P. Dos Santos , and K. V. Bloch , “Prevalence of Self‐Reported Gingival Bleeding in a Representative Sample of the Brazilian Adolescent Population,” Journal of Clinical Periodontology 45, no. 8 (2018): 952–958, 10.1111/jcpe.12959. 29904930

[17] M. J. Batista , L. B. Rihs , C. D. S. Gonçalo , F. M. M. Kubo , R. C. D. Amaral , and M. D. L. R. D. Sousa , “Treatment Needs and Self‐Perception of Oral Health Among Adolescents,” RGO ‐ Revista Gaúcha de Odontologia 60, no. 3 (2012): 289–296.

[18] M. T. Keboa , A. S. Madathil , and B. Nicolau , “Perceived and Assessed Dental Treatment Needs of Schoolchildren in Benoe Division, Cameroon,” JDR Clinical & Translational Research 4, no. 2 (2019): 160–166, 10.1177/2380084418814485. 30931712

[19] S. Krisdapong , P. Prasertsom , K. Rattanarangsima , and A. Sheiham , “Associations Between Perceived Needs for Dental Treatment, Oral Health‐Related Quality of Life and Oral Diseases in School‐Aged Thai Children,” Community Dentistry and Oral Epidemiology 42, no. 4 (2014): 323–332, 10.1111/cdoe.12092. 24428381

[20] K. O. Mashoto , A. N. Astrøm , J. David , and J. R. Masalu , “Dental Pain, Oral Impacts and Perceived Need for Dental Treatment in Tanzanian School Students: A Cross‐Sectional Study,” Health and Quality of Life Outcomes 7 (2009): 73, 10.1186/1477-7525-7-73. 19643004 PMC2726126

[21] D. R. Figueiredo , J. L. Bastos , and K. G. Peres , “Association of Adverse Oral Health Outcomes With Socioeconomic Inequalities and Dental Needs in Brazilian Adolescents,” Cadernos de Saúde Pública 33, no. 5 (2017): e00165415, 10.1590/0102-311X00165415. 28640329

[22] Brasil. Ministério da Saúde (MS). Projeto SB Brasil , Condições de Saúde Bucal da População Brasileira, 2002–2003: Resultados Principais (MS, 2004).

[23] World Health Organization (WHO) and Global Youth Tobacco Survey Collaborative Group , Global Youth Tobacco Survey (GYTS): Core Questionnaire With Optional Questions, Version 1.0 (WHO, 2012).

[24] Instituto Brasileiro de Geografia e Estatística (IBGE). Pesquisa Nacional de Saúde do Escolar (IBGE, 2016).

[25] A. Sheiham and R. G. Watt , “The Common Risk Factor Approach: A Rational Basis for Promoting Oral Health,” Community Dentistry and Oral Epidemiology 28, no. 6 (2000): 399–406, 10.1034/j.1600-0528.2000.028006399.x. 11106011

[26] N. G. Nery , J. L. F. Antunes , L. M. R. Jordão , and M. D. C. M. Freire , “Can the School Environment Influence Oral Health‐Related Behaviours? A Multilevel Analysis of the Brazilian National Adolescent School‐Based Health Survey 2015,” Community Dentistry and Oral Epidemiology 49, no. 1 (2021): 23–32, 10.1111/cdoe.12569. 32815223

[27] M. W. Heft , G. H. Gilbert , B. J. Shelton , and R. P. Duncan , “Relationship of Dental Status, Sociodemographic Status, and Oral Symptoms to Perceived Need for Dental Care,” Community Dentistry and Oral Epidemiology 31 (2003): 351–360, 10.1034/j.1600-0528.2003.00014.x. 14667006

[28] C. J. Moores , S. A. M. Kelly , and P. J. Moynihan , “Systematic Review of the Effect on Caries of Sugars Intake: Ten‐Year Update,” Journal of Dental Research 101, no. 9 (2022): 1034–1045, 10.1177/00220345221082918. 35302414

[29] A. C. L. T. Massoni , É. Porto , L. R. B. O. Ferreira , et al., “Access to Oral Healthcare Services of Adolescents of a Large‐Size Municipality in Northeastern Brazil,” Brazilian Oral Research 34 (2020): e029, 10.1590/1807-3107bor-2020.vol34.0029. 32236318

[30] E. P. Fonseca , E. F. Ferreira , M. H. N. G. Abreu , A. C. Palmier , and A. M. D. Vargas , “Relação Entre Condição Gengival e Fatores Sociodemográficos de Adolescentes Residentes em Uma Região Brasileira,” Ciência & Saúde Coletiva 20, no. 11 (2015): 3375–3384, 10.1590/1413-812320152011.00142015. 26602715

